# Decreased work productivity due to primary palmar hyperhidrosis. What is the cost?

**DOI:** 10.1002/ski2.24

**Published:** 2021-04-05

**Authors:** J. K. Kristensen, C. Nielsen

**Affiliations:** ^1^ Hyperhidrosis Skin and Hyperhidrosis Clinic Copenhagen Denmark

## Abstract

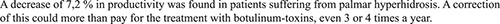

A decrease of 7,2 % in productivity was found in patients suffering from palmar hyperhidrosis. A correction of this could more than pay for the treatment with botulinum‐toxins, even 3 or 4 times a year.

1

1

Dear Editor,

Primary hyperhidrosis is a chronic, idiopathic condition characterised by excessive sweat production beyond physiologic needs. Hyperhidrosis is said by patients to impair their work productivity. This problem has not been addressed before as far as palmar hyperhidrosis is concerned. We therefore set out to investigate the issue using a validated tool, the Work Limitation Questionnaire (WLQ). This has not been tried in palmar hyperhidrosis previously.

Consecutive patients seeking treatment for palmar hyperhidrosis and often also seeking treatment for plantar and axillary hyperhidrosis were enrolled. They answered the WLQ as well as the Dermatology Life Quality Index (DLQI) and the Hyperhidrosis Disease Severity Scale (HDSS), before treatment.

A large number of instruments exist to measure health‐related productivity changes. Systematic reviews^1^ point to the WLQ as a reliable tool. This tool is validated extremely cautiously and a translation to Danish has been performed and also validated thoroughly. The questionnaire was obtained from Mapi Research.

The WLQ was developed by Lerner and associates,^2^ ^,^ ^3^ and the development was described in multiple papers. It is an easy‐to‐use questionnaire, measuring the degree to which employed individuals are experiencing limitations on the job due to their health problems and measure health‐related productivity loss.

In primary palmar hyperhidrosis, a substantial impairment of the occupational, emotional and physical status of affected individuals has been described.^4^, ^5^ Palmar hyperhidrosis is quite common, and in some populations, more than 2% are sufferers.^6^, ^7^ Presenteeism, the decline in on‐the‐job productivity, resulting from worker illness, is a significant yet hidden cost to employers. Presenteeism has been associated with chronic medical conditions ranging from allergies to osteoarthritis. In this context we will measure presenteeism, (productivity loss) in patients suffering from hyperhidrosis of the hands.

This small study was planned to be a feasibility study. Patients were consecutively enrolled in December 2020. Only 23 patients were recruited as our employment at Empano (a private clinic specialising in hyperhidrosis) terminated by the end of December. One patient was not included as she was 68 and had left employment. The patients completed the WLQ which is a 25‐item instrument that measure employees experience of work limitations and productivity losses due to their health problems during the last 2 weeks. Patients rate their ability to perform tasks on four limitation scales: Time Management, Physical Demands, Mental and Interpersonal Demands and Output Demands. Responses for each are summed and reported as a scale score of 0–100 (not limited–limited all the time). Transformed, scale scores, indicate the percentage of time that patients were limited in performing a specific dimension of work in the prior 2 weeks, and as such, estimates health‐related deficits in work performance. The WLQ index is the weighted sum of the scores from the four WLQ scales and estimates overall health‐related productivity loss. The index score is interpreted as the percentage reduction in health‐related productivity compared to a benchmark group of employees with no work limitations. From the WLQ index, percentage productivity loss can be calculated. A technical report on development and calculation of values can be obtained from Tufts Medical Centre. Formulaes for transforming scale scores to index scores and finally to productivity loss in percent, can be found in this material. It is not necessary to adjust scores by age, gender or other demographic characteristics.

Of 23 consecutive patients enrolled in this study all were young working people. Ten, were students (one a medical student). Twelve were placed in the International Standard Classification of Occupations‐08 skill level 2 (clerical and sales work) and one finally in skill level 4 (a lawyer). Due to the small number of patients, we did not try to correlate presenteeism with skill levels. All our patients were suffering from manifest hyperhidrosis with HDSS 3 or 4.

The correlation coefficient for the relation between DLQI and the index score, were calculated. Mean and standard deviations were calculated.

We did not eliminate patients that stated they at present, were attending courses or schooling periods. Also, we were aware that working at home during the COVID‐pandemic was common, and partly or completely changed the workplace situation**.**

From Table 1 a few demographic data as well as the results of DLQI, HDSS and WLQ% can be seen.

**TABLE 1:** Study results

Gender	Age	HDSS	DLQI	WLQ %
F	25	3	9	5.48
M	33	3	6	3.90
F	27	3	14	0.70
M	30	4	20	11.00
F	23	3	8	4.20
F	24	4	9	9.00
F	19	3	9	2.60
M	19	3	6	1.23
M	22	4	11	11.10
M	24	4	16	12.12
F	26	3	11	8.30
F	35	3	14	0.70
F	25	3	11	10.30
M	32	4	11	0.24
F	24	3	5	3.90
F	31	4	18	7.00
F	24	4	9	6.30
F	25	4	15	23.60
F	24	4	14	9.10
F	20	3	14	16.40
M	34	4	16	7.70
F	27	3	8	4.60
F	52	4	5	7.10
Mean	27.17		11.26	7.24
SD	7.07		4.20	5.45

*Note:* Pearsons *r* (coefficient of correlation). DLQI versus WLQ% = 0.4249 (*p* < 0.05).

Abbreviations: DLQI, Dermatology Life Quality Index; HDSS, Hyperhidrosis Disease Severity Scale; WLQ, Work Limitation Questionnaire.

We have not presented the four dimensions of WLQ as we could not find any difference between the dimensions. We therefore must conclude that all four dimensions were important. Not only ‘time management’ or ‘output demands’.

WLQ% is computed from the WLQ index and is a work productivity percentage loss.

The mean productivity loss is 7.24%. As the mean pretax income in the age group is 338.122 Danish Kroner the loss in productivity can be transformed to 24.480 Danish Kroner a year. At an exchange rate of 0.11973, this equals 2.931 British Pounds.

A hypothetical gain in productivity is then able to, more than pay for the treatment.

Indirect costs of productivity loss, clearly exceed, the total direct cost in psoriasis.^8^ The same is true for hyperhidrosis. There is proof here that intervention can, potentially, reduce health‐related productivity loss and improve patients' quality of life. Savings from increased work productivity might offset comparatively high acquisition costs of botulinum toxins. Regaining productivity, could easily pay for 3–4 treatments a year, using botulinum toxin. This could be even more than needed.

A few years ago, it was shown that regarding axillary hyperhidrosis cost effectiveness equalled, after 13.3 years, when comparing botulinum toxin to sympathectomy.^9^ It is to be expected that the same would be true for palmar hyperhidrosis, but to our knowledge, this issue has not been studied.

Due to cost effectiveness and small number of side effects botulinum toxin will probably be preferred to sympathectomy in the future, taking in account that almost 90% of patients suffer from compensatory hyperhidrosis after surgery. Unfortunately, the effectiveness of botulinum toxin for palmar hyperhidrosis is not yet supported by science, as the effectiveness has not been scrutinised in a controlled manner.

Finally taking a look at a treatment algorithm, we agree that local treatment and iontophoresis should be tried first. But after that, it is time for botulinum toxin, which in palmar hyperhidrosis often is a combination of type A and type B, in an effort to avoid problems with grip function.

In rare cases where this does not work well enough, you can add anticholinergics. Should all of this, not have sufficient effect, in rare cases, you might consider sympathectomy.^10^

## LIMITATIONS

A small number of patients, patients were all young, Corona isolation forced many to work from home, manual labourers, was not represented.

## CONFLICT OF INTERESTS

The authors declare that there are no conflict of interests.

## Data Availability

The full data is available from the corresponding author.

## References

[1] Noben CYG , Evers SMAA , Nijhuis FJ , de Rijk AE . Quality appraisal of generic self‐reported instruments measuring health related productivity changes: a systematic review. BMC Publ Health. 2014;14:115–36. 10.1186/1471-2458-14-115 PMC3942271 24495301

[2] Lerner D , Amick BC , Lee JC , Rooney T , Rodgers WH , Chang H , et al. Relationship of employee‐reported work limitations to work productivity. Med Care. 2003;41:649–59. 12719689 10.1097/01.MLR.0000062551.76504.A9

[3] Lerner D , Adler DA , Chang H , Berndt ER , Irish JT , Lapitsky L , et al. The clinical and occupational correlates of work productivity loss among employed patients with depression. J Occup Environ Med. 2004;46:S46–55. 15194895 10.1097/01.jom.0000126684.82825.0a PMC4283812

[4] Kristensen JK , Möller S , Vestergaard DG , Horsten HH , Swartling C , Bygum A . Anxiety and depression in primary hyperhidrosis: an observational study of 95 consecutive Swedish outpatients. Acta Derm Venereol. 2020;100: adv 00240. 10.2340/00015555-3598 PMC9207642 32725249

[5] Kristensen JK , Vestergaard DG , Swartling C , Bygum A . In hyperhidrosis, quality of life is even worse than in acne, eczema, or psoriasis. A comparison of Skindex‐16 and Dermatology Life Quality Index (DLQI). Int J Dermatol. 2020. 10.1111/ijd.15164 32880921

[6] Lai F‐C , Tu Y‐R , Li Y‐P , Li X , Lin M , Chen J‐F , et al. Nationwide epidemiological survey of primary palmar hyperhidrosis in the People's Republic of China. Clin Auton Res. 2015;25:105–8. 25381140 10.1007/s10286-014-0259-5

[7] Lerner D , Rodgers WH , Chang H . Confidential: scoring the Work Limitation Questionnaire (WLQ). Technical report, Obtainable through Tufts Medical Center. 2009.

[8] Schmitt JM , Ford DE . Work limitation and productivity loss are associated with health‐related quality of life but not with clinical severity in patients with psoriasis. Dermatology. 2006;213:102–10. 16902286 10.1159/000093848

[9] Gregoriou S , Sidiropoulou P , Kontochristopoulos G , Rigopoulos D . Management strategies of palmar hyperhidrosis: challenges and solutions. Clin Cosmet Investig Dermatol. 2019;12:733–44. 10.2147/CCID.S210973 PMC6781850 31632121

[10] Gibbons JP , Nugent E , O'Donohoe N , Feeley M . Experience with botulinum toxin therapy for axillary hyperhidrosis and comparison to modelled data for endoscopic thoracic sympathectomy—A quality of life and cost effective analysis. Surgeon. 1016;14:260–4. 10.1016/j.surge.2015.05.002 26071930

